# SENSORY PROCESSING DURING CHILDHOOD IN PRETERM INFANTS: A SYSTEMATIC REVIEW

**DOI:** 10.1590/1984-0462/;2017;35;1;00008

**Published:** 2017-02-20

**Authors:** Ana Carolina Cabral de Paula Machado, Suelen Rosa de Oliveira, Lívia de Castro Magalhães, Débora Marques de Miranda, Maria Cândida Ferrarez Bouzada

**Affiliations:** a Faculdade de Medicina da Universidade Federal de Minas Gerais (UFMG), Belo Horizonte, MG, Brasil.; b Faculdade de Educação Física da UFMG, Belo Horizonte, MG, Brasil.

**Keywords:** Sensation disorders, Infant, premature, Review

## Abstract

**Objective:**

To conduct a systematic search for grounded and quality evidence of sensory processing in preterm infants during childhood.

**Data source:**

The search of the available literature on the theme was held in the following electronic databases: Medical Literature Analysis and Retrieval System Online (Medline)/PubMed, Latin American and Caribbean Literature in Health Sciences (Lilacs)/Virtual Library in Health (BVS), Índice Bibliográfico Español de Ciencias de la Salud (IBECS)/BVS, Scopus, and Web of Science. We included only original indexed studies with a quantitative approach, which were available in full text on digital media, published in Portuguese, English, or Spanish between 2005 and 2015, involving children aged 0-9years.

**Data synthesis:**

581 articles were identified and eight were included. Six studies (75%) found high frequency of dysfunction in sensory processing in preterm infants. The association of sensory processing with developmental outcomes was observed in three studies (37.5%). The association of sensory processing with neonatal characteristics was observed in five studies (62.5%), and the sensory processing results are often associated with gestational age, male gender, and white matter lesions.

**Conclusions:**

The current literature suggests that preterm birth affects the sensory processing, negatively. Gestational age, male gender, and white matter lesions appear as risk factors for sensoryprocessing disorders in preterm infants. The impairment in the ability to receivesensory inputs, to integrateand to adapt to them seems to have a negative effect on motor, cognitive, and language development of these children. We highlight the feasibility of identifying sensory processing disorders early in life, favoring early clinical interventions.

## RESUMO

**Objetivo:**

Buscar sistematicamente evidências fundamentadas e de qualidade sobre o processamento sensorial em crianças nascidas pré-termo no período da infância.

**Fontes de dados:**

A busca da literatura disponível sobre o tema foi realizada nas bases de dados eletrônicas Sistema *Online* de Busca e Análise de Literatura Médica (Medline)/PubMed, Literatura Latino-Americana e do Caribe em Ciências da Saúde (Lilacs)/Biblioteca Virtual em Saúde (BVS), Índice Bibliográfico Español de Ciencias de la Salud (IBECS)/BVS, Scopus e Web of Science. Foram incluídos estudos indexados, originais, quantitativos e disponíveis na íntegra em meio digital, publicados em português, inglês ou espanhol, entre 2005 e 2015, envolvendo crianças entre zero e 9 anos de idade.

**Síntese dos dados:**

A busca identificou 581 artigos, e oito foram incluídos conforme critérios de elegibilidade. Destes, seis estudos (75%) encontraram alta frequência de processamento sensorial alterado em crianças pré-termo. A associação entre processamento sensorial e desfechos de desenvolvimento foi observada em três estudos (37,5%). Verificou-se associação entre processamento sensorial e características neonatais em cinco estudos (62,5%). Os resultados de processamento sensorial frequentemente se associam a idade gestacional, sexo masculino e lesões da substância branca.

**Conclusões:**

Análise da literatura atual sugere que prematuridade tem impacto negativo no processamento sensorial. Idade gestacional, sexo masculino e lesões de substância branca aparecem como fatores de risco para alterações de processamento sensorial em crianças nascidas pré-termo. O prejuízo na capacidade de receber informações sensoriais, de integrar e de adaptar-se a elas parece interferir negativamente no desenvolvimento motor, cognitivo e de linguagem dessas crianças. Destaca-se a viabilidade da identificação das alterações de processamento sensorial nos primeiros anos de vida, favorecendo o encaminhamento precoce para intervenções clínicas.

## INTRODUCTION

Sensory processing concerns the way the central nervous system manages the information received from sensory organs, that is, the visual, auditory, tactile, gustatory, olfactory, proprioceptive, and vestibular stimuli. The process includes reception, modulation, integration, discrimination, and organization of sensory stimuli as well as adaptive behavioral responses to these stimuli.^1^

Sensory processing disorder (SPD) is the term used to refer to difficulties in processing and using sensory information for the regulation of physiological, motor, affective, and/or attention responses that interfere in the organization of behavior and in the participation in activities of daily living.^2^ ^,^ ^3^SPD can be observed in individuals without any apparent clinical condition, but usually occurs associated with other diagnoses, such as autism spectrum disorder, attention deficit disorder/hyperactivity, developmental coordination disorder, and fragile X syndrome.^4^ ^,^ ^5^ Its prevalence is estimated from 5 to 16% in apparently normal population and 30 to 80% among the population with specific diagnoses.^6^

Although the etiology of SPD remains unknown, genetic, family, and environmental factors have been reported in the literature.^3^ ^,^ ^7^ In this context, preterm infants (born before 37 weeks of gestation) are considered at risk for SPD. This risk is a consequence of both the interruption of neurobiological intrauterine development and the sensory experiences of the Neonatal Intensive Care Unit (NICU) environment, which can alter the development and functioning of the sensory systems.^8^ Although there is evidence of sensory processing disorders in children who were born preterm, there are still relatively few studies on the association of sensory processing with prematurity, hindering the general view of the prevalence and persistence of the symptoms of SPD in this population.

### Theoretical framework

SPD is an alteration of development in the first childhood, that has received increasing attention in the last decade.^9^ ^,^ ^10^ ^,^ ^11^ ^,^ ^12^ ^,^ ^13^ ^,^ ^14^ Alterations in responses to sensory stimuli were first identified as a clinical condition by the American occupational therapist Anna Jean Ayres in 1972, when she was studying children with learning difficulties.^4^ By associating knowledge on neurobiology with detailed observation of children’s behavior, Ayres theorized that the impairment of sensory processing can lead to various functional problems, and she named this condition as sensory integration dysfunction.^15^ Currently, the clinical characteristics described by Ayres is called SPD.^16^ This change in nomenclature was proposed by Miller et al.^15^ based on the argument that the use of the term “sensory integration,” as adopted by Ayres, often refers to a neurophysiological cellular process and not to the behavioral responses to sensory stimuli. Therefore, the new terminology becomes more suitable as it distinguishes the clinical condition, which characterizes subjects with atypical behavioral responses to sensory stimulation, from the neurophysiological cellular process involved.^15^

SPD is a heterogeneous condition that includes several subtypes.^5^ Miller et al.^15^ characterized three classic SPD subtypes: sensory modulation disorder, sensory discrimination disorder, and sensory-based motor disorder. The first involves the difficulty to transform sensory information into behaviors that are consistent with the intensity and nature of the sensory experience. Its symptoms include over-responsivity (more intense responses, faster or longer lasting than those normally observed), under-responsivity (less intense responses or slower than typically observed), and sensory craving (intense and insatiable desire for sensory stimuli). Sensory discrimination disorder refers to the difficulty in discriminating sensory stimuli qualities, resulting in reduced ability to detect similarities and differences between stimuli, and to differentiate the temporal and spatial qualities of the perceived stimulus. Children with this type of disorder perceive stimuli and regulate their responses, but are not capable of identifying the exact nature of the stimulus or its precise location. Finally, sensory-based motor disorder is the difficulty in stabilizing the body (postural disorder) or plan and sequence coordinated movements (dyspraxia) based on sensory information.^15^

It is worth mentioning that none of these conditions is in the Diagnostic and Statistical Manual of Mental Disorders (DSM-V) or in the International Statistical Classification of Diseases and Related Health Problems (ICD-10), and there is no consensus on howthey should be defined.^14^ However, some are addressed, directly or indirectly, in two reference manuals for diagnostic development classification: the Diagnostic Manual for Infancy and Early Childhood from the Interdisciplinary Council on Developmental and Learning Disorders (ICDL), and the Diagnostic Classification of Mental health and Developmental Disorders of Infancy and Early Childhood-revised (DC 0-3R).^6^ ^,^ ^9^ ^,^ ^15^

The clinical manifestations of SPD are varied and include crying and excessive agitation, difficulty in self-consoling, sleep problems and acceptance of food, exacerbation of parental separation anxiety, persistent and exaggerated shyness with strangers, intolerance to change, lack of interest and indifference in social interaction.^16^ ^,^ ^17^ Functional problems commonly associated with SPD in early childhood include decreased social skills and participation in games, reduced frequency, duration, and complexity of adaptive responses; impaired self-confidence and/or self-esteem; and poor motor skills.^18^ Problems in balance, gross and fine motor coordination, and motor planning, as well as delayed language acquisition, tactile hypersensitivity, or severe dispersion become evident in preschool. At school, problems with writing and reading, attention deficit, emotional difficulties, and poor interaction with colleagues arise.^19^ These problems may persist in adulthood, resulting in social and emotional difficulties.^18^

The diagnosis of SPD is based on child behavior observation and/or the application of questionnaires to parents.^20^ Although they are not yet validated in Brazil, there are several assessment tools to identify SPD such as Infant/Toddler Sensory Profile,^21^ Sensory Integration and PraxisTest,^22^ Sensory Profile,^23^ DeGangi-Berk Test of Sensory Integration,^24^ Observations Based on Sensory IntegrationTheory,^25^ Test of Sensory Functions in Infants,^26^ and Sensory Rating Scale.^27^

### Sensory processing disorders and prematurity

Recent studies show that children who were born prematurely have different responses to sensory stimuli and may exhibit alterations in sensory processing.^28^ ^,^ ^29^ ^,^ ^30^These differences may be explained by two factors that seem to interact: the cumulative effect of medical complications associated with premature birth (periventricular leukomalacia, severe intraventricular hemorrhage, sepsis, low growth, bronchopulmonary dysplasia, and use of postnatal steroids) and sensory experiences in the NICU environment in the early stages of development.^30^ ^,^ ^31^

Environmental stimuli of the fetus and child after birth, including sound, voice, touch, movement, smell, and vision experiences are crucial for the proper development of the sensory systems. Although not all these experiences have a significant role in establishing the initial patterns of the sensory systems connectivity, they contribute to the improvement and maintenance of appropriate connections in the developing brain.^32^

According to Lickliter,^33^ sensory abilities of the fetus and the context of development in the uterus effectively limit and regulate the amount, type, and duration of sensory stimulation that occurs during the prenatal period. This pattern of sensory stimulation is radically altered by preterm birth, with significant changes in normal patterns of tactile, vestibular, proprioceptive, olfactory, auditory, and visual stimulations.^33^ For example, the newly born preterm in the NICU receives smaller quantities of tactile-vestibular stimulation resulting from maternal movement, but there are significant increase in other types of stimulation that are not present in the intrauterine environment (bright lights, high noise levels, excessive handling, and frequent painful interventions). This reality can have lasting effects on the developing brain and interfere in the natural development of sensory systems.^31^

In this scenario, the objective of this review was to search for grounded and quality evidence concerning the sensory processing in preterm infants. This was conducted by means of a systematic knowledge synthesis,based on the literature available on the subject that was published between 2005 and 2015.

## METHOD

A systematic literature review was carried out following the protocol adapted from the principles established by the Cochrane Library.^34^ Inclusion criteria for the selection of studies were defined as well as data sources for the research, target audience, time limit, descriptors and free terms, synthesis, and interpretation of results.

Only studies that were indexed, original, quantitative, and fully available in digital media were included in the review. They were also published in Portuguese, English, or Spanish between August 2005 and August 2015, and included children in the age group of zero to nine years. This time limit was established aiming at reviewing the most recent literature on the subject, and the selection of the age range is due to the particular interest in investigating how preterm birth can affect sensory processing during childhood.

First, an electronic search was conducted in the databases Medical Literature Analysis and Retrieval System Online (Medline)/PubMed, *Literatura Latino-Americana e do Caribe em Ciências da Saúde* (Lilacs)/*Biblioteca Virtual em Saúde* (BVS), and *Índice Bibliográfico Español de Ciencias de la Salud* (IBECS)/BVS, based on the strategy of the combination of two sets of keywords using the boolean operators AND and OR. The descriptors chosen were “sensation disorders” and “infant, premature”(in consultation with Health Sciences Descriptors - DeCS). Free terms were “prematurity,” “sensory processing disorders,” “sensory functions in infants,” “sensory profile of infants,” and their counterparts in Portuguese and Spanish. We opted for the use of descriptors and free terms in the strategy design to ensure a more comprehensive search, reducing the risk of missing relevant studies.

Reading of articles’ abstracts that were located in the search was the main source of selection of the publications, which intended to confirm that they were related to the theme and met the established inclusion criteria. In addition to the electronic search, the citation search was carried out using the reference lists of the selected articles and their citation indexes in Scopus and Web of Science databases. All the search process was conducted by the first and second authors. The two reviewers read and selected the papers independently, and then they did across-check. Finally, the matching articles were selected. The retrieval of the selected papers in digital media in their full version was performed in the Capes Portal.

Full reading of the articles enabled the extraction of the relevant aspects of each article and its summarization in tables to ensure that the same information was obtained from all publications. This step was conducted by the first and second authors, and the results were subsequently discussed and analyzed together with the other authors.

## RESULTS

By the combination of descriptors and free terms mentioned earlier, 581 articles were found in the electronic search. Six articles were selected after the application of the established inclusion criteria. The significant difference between the number of publications found and the number of publications selected is due to the fact that most studies covered different populations from the target defined for this investigation. Studies involving individuals with autism spectrum disorder and attention deficit disorder/hyperactivity, and those with children born at term were excluded. Other studies on sensorineural impairments (blindness and deafness), retinopathy of prematurity, asthma, and drug effects, studies using animals, literature reviews and others publications before the established cutoff date were also excluded. Citation searching enabled the selection of two additional articles.

The eight selected publications were included in the study.­^35^ ^,^ ^36^ ^,^ ^37^ ^,^ ^38^ ^,^ ^39^ ^,^ ^40^ ^,^ ^41^ ^,^ ^42^General characteristics of each study, such as author, year of publication, country where the study was conducted, and study design are presented in Table 1. The relevant information concerning the characteristics of the samples, the age range, the objectives, and assessment tools are summarized in Table 2, and the main results of the papers reviewed are described in Table 3.

**Table 1:** General characteristics of the studies included in the systematic reviewof the literature on sensorial processing in preterm children, which were published between 2005 and 2015.

Author, year	Country	Study design
Rahkonen et al.,^35^2015	Finland	Prospective
Adams et al.,^36^2015	United States of America	Cross-sectional
Cabral et al.,^37^2015	Brazil	Cross-sectional
Chorna et al.,^38^2014	United States of America	Prospective
Eeles et al.,^39^2013	Australia	Prospective
Eeles et al.,^40^2013	Australia	Cross-sectional
Wickremasinghe et al.,^41^2013	United States of America	Cross-sectional
Bart et al.,^42^2011	Israel	Cross-sectional

**Table 2:** Relevant information on the samples studied, objectives, age, assessments, and instruments applied in the studies included in the systematic review of literature on sensory processing in preterm infants, which were published between 2005 and 2015.

Author	Sample	Objectives	Age,assessments, and instruments
Rahkonen et al.^35^	44 preterm infants (GA <28 weeks)	- To investigate the SP and its association with neonatal risk factors, neuroanatomical data and neurodevelopment	- Age term: brain neuroanatomical assessment (MRI) - Two years of corrected age: evaluation of SP (ITSP); neurological assessment (Hempel); assessment of mental development (GMDS); assessment of cognition and language (BSID)
Adams et al.^36^	54 preterm infants (GA <34 weeks) and 73 full terminfants (GA>37 weeks)	- To compare the SPof preterm and full term infants and to check the association of SPwith executive and adaptive functions in preterm infants	- Between 3 and 5 years old: evaluation of SP (SSP); assessment of executive function (BRIEF-P and performance in six interactive tasks); evaluation of adaptive function (Vineland-II)
Cabral et al.^37^	15 preterm infants (GA <37 weeks) and 15 full term infants (37> GA>42 weeks)	- To compare and investigate the association between SP and motor development in preterm and full terms infants	- Between 4 and 6 months of corrected age: evaluation of SP (TSFI); evaluation of motor development by AIMS.
Chorna et al.^38^	40 preterm infants (GA <30 weeks)	- To assess the outcome of SP and its association with neurodevelopment	- Corrected age of 4-12 months: evaluation of SP (TSFI) - Two years of corrected age: evaluation of cognition, motor skills,and language (BSID).
Eeles et al.^39^	253 preterm infants (GA <32 weeks) and 65 full term infants (GA>36 weeks)	- To examine the SP and its environmental and biological influences	- Age at full term: neuroanatomical assessment (MRI) - Two years of corrected age: evaluation of SP (ITSP)
Eeles et al.^40^	241 preterm infants with GA<30 weeks	- To examine the SP and its association with neurodevelopment	- At two years of corrected age: evaluation of SP (ITSP) and neurodevelopment (BSID)
Wickremasinghe et al.^41^	107 preterm infants (GA≤32 weeks)	- To check the outcome of SP and its association with neurodevelopment	- Between 1-8 years: evaluation of SP (ITSP and SSP); evaluation of neurodevelopment (BSID, WPPSI, and WISC)
Bart et al.^42^	124 preterm infants (34> GA<36 weeks and 33 full term infants (GA>37 weeks)	- To compare the SP and participation in daily activities and to verify the association of SP with neonatal characteristics in preterm infants	- One year of chronological age: evaluation of SP (ITSP and TSFI) and of the participation in daily activities (own questionnaire).

GA: gestational age; SP: sensory processing; MRI: magnetic resonance imaging; ITSP: Infant/Toddler Sensory Profile; GMDS: Griffiths Mental Developmental Scales; BSID: Bayley Scales of Infant and Toddler Development; SSP: Short Sensory Profile; BRIEF-P: Behavior Rating Inventory of Executive Function - Preschool Version; Vineland-II: Vineland Adaptive Behavior Scales, Second Edition, Parent/Caregiver Rating Form; TSFI: Test of Sensory Function in Infants; AIMS: Alberta In fant Motor Scale; WPPSI: Wechsler Preschool and Primary Scale of Intelligence; WISC: Wechsler Intelligence Scale for Children.

**Table 3:** Main results of the studies included in the systematic review of the literature on sensory processing in preterm infants, which were published between 2005 and 2015.

Author	Main results
Rahkonen et al.^35^	There was a high frequency of altered SP. SP outcome was associated with white matter lesions and surgical closure of patent ductus arteriosus. No association was found between SP and neurodevelopment.
Adams et al.^36^	Preterm infants had significant lower scores on SSP compared to full term infants, with higher frequency of altered sensory processing. GA was associated with SP. Association was found between SP and executive function in preterm infants. There was no association between SP and adaptive function in preterm infants.
Cabral et al.^37^	Preterm infants are different from their full term peers in relation to the SP, especially regarding the reactivity to deep tactile pressure. No difference was found between the groups with regard to motor development. There was no statistically significant association of SP with motor development in both groups.
Chorna et al.^38^	There was a high frequency of abnormal SP, especially regarding reactivity to vestibular stimulation and reactivity to deep tactile pressure. GA, male gender, white matter lesions, and caregiver education were associated with SP outcomes. SP was associated with motor and language development.
Eeles et al.^39^	Preterm infants showedhigher frequency of SPalterations when compared to peers at term. Male gender, white matter lesions, and hospital stay were associated with SP outcomes.
Eeles et al.^40^	An association of SP outcomes with motor, cognitive, and languagedevelopment was found.
Wickremasinghe et al.^41^	There was a high frequency of altered SP, with similar prevalence at age ranges 1-4 years and 4-8 years. The most affected areas were the auditory, vestibular, and tactile processing. No association was observed between SP, neonatal characteristics, and neurodevelopment.
Bart et al.^42^	Preterm infants are different from the peers born at term in relation to the SP. There was a significant difference between groups in the frequency of participation in all areas of activities, except for leisure. The groups were also different with respect to the satisfaction of parents, except for social participation. Association was found between SP, GA, and head circumference. Days at hospital and multiple births were predictors of participation in daily activities and parental satisfaction.

SP: sensory processing; SSP: Short Sensory Profile; GA: gestational age.

All the studies included in this review were published in the last five years, and only one was carried out in Brazil(12.5%).^37^ The others were developed in foreign countries, more frequently (37.7%)^36^ ^,^ ^38^ ^,^ ^41^ in the United States of America. Five studies (62.5%)^36^ ^,^ ^37^ ^,^ ^40^ ^,^ ^41^ ^,^ ^42^ are cross-sectional and three studies (37.5%)^35^ ^,^ ^38^ ^,^ ^39^ are prospective.

Of the studies, six (75%)^35^ ^,^ ^36^ ^,^ ^38^ ^,^ ^39^ ^,^ ^40^ ^,^ ^41^investigated children who were born with gestational age below 34 weeks. The sample size of the studies ranged from 15 to 253 children who were born preterm.

With regard to the age in which sensory processing was evaluated, six studies (75%)^35^ ^,^ ^37^ ^,^ ^38^ ^,^ ^39^ ^,^ ^40^ ^,^ ^41^encompassed the two first years of life. The Infant/Toddler Sensory Profile^21^ was used as the evaluation tool in five studies (50%),^35^ ^,^ ^39^ ^,^ ^40^ ^,^ ^41^ ^,^ ^42^ and the Test of Sensory Function in Infants^26^ was applied in three studies (37.5%);^37^ ^,^ ^38^ ^,^ ^42^ in one study (12.5%),^42^ these two instruments were applied simultaneously. In one study, authors (12.5%),^36^ chose to evaluate the sensory processing in the period between 3 and 5 years of age and to apply the Short Sensory Profile.^23^ In another paper (12,5%),^41^ the evaluation was carried out in period from 1 to 8 years of age, and the Infant/Toddler Sensory Profile^21^ was applied in the first two years of life, and the Short Sensory Profile^23^ was used from the third year on.

The comparison of children who were born prematurely with their peers who were born at term was carried out in four studies(50%),^36^ ^,^ ^37^ ^,^ ^39^ ^,^ ^42^ whose results confirm that the two groups of children present differences in relation to the sensory processing, and that preterm infants have higher frequency of alterations in sensory processing. The other four studies (50%)^35^ ^,^ ^38^ ^,^ ^40^ ^,^ ^41^ presented a comparison with normative data established by the instruments and also found high frequency of alterations in sensory processing in preterm children.

To verify the association of sensory processing with development outcomes,was the objective of six studies(75%).^35^ ^,^ ^37^ ^,^ ^38^ ^,^ ^40^ ^,^ ^41^ This association was observed in three of them(37.5%).^36^ ^,^ ^38^ ^,^ ^40^ Six studies(75%)^35^ ^,^ ^36^ ^,^ ^38^ ^,^ ^39^ ^,^ ^41^ ^,^ ^42^ analyzed the association of sensory processing with neonatal characteristics, which was not found in only one study(12.5%).^41^ Gestational age and male gender appear to be risk factors, as they are often associated with sensory processing results. The association with neuroanatomical data, which was obtained by magnetic resonance imaging (MRI), was investigated in two studies (25%),^35^ ^,^ ^39^ and in both of them an association of poorer results of sensory processing with white matter lesions was found.

## DISCUSSION

Analysis of the selected literature suggests that preterm infants are different from their peers who were born at term with respect to sensory processing. They present high frequency of alterations in sensory processing during childhood. Therefore, prematurity can be considered a risk factor for SPD. This fact is not surprising, as preterm newborns, in addition to being biologically vulnerable, are deprived of the natural sensations of the intrauterine environment and need prolonged periods of stay in the NICU, where the sensory experience conflicts with their needs. 

Most studies investigated the sensory processing of preterm children who were born with gestational age below 34 weeks; however, the study of Bart et al.^42^ is important to highlight as it investigated the effect of late prematurity (birth between 34 and 36 weeks of gestation) on sensory processing in children in the first year of life. Its results showed that late preterm infants are different from their peers who were born at term with respect to the sensory processing, suggesting that the late prematurity may also be associated with sensory processing issues. Further studies with larger samples are needed to investigate this issue.

From the six studies that were reviewed, the sensory processing was evaluated in the first two years of life,^35^ ^,^ ^37^ ^,^ ^38^ ^,^ ^39^ ^,^ ^40^ ^,^ ^42^ thereby leading to the conclusion that SPD signals can be identified early. Early identification of SPD is considered crucial because children can benefit from clinical interventions to improve their sensory capacities and reduce the negative impact of sensory processing difficulties on their development.^2^ ^,^ ^43^ ^,^ ^44^ ^,^ ^45^

With regard to the evaluation instruments, Infant/Toddler Sensory Profile (ITSP)^21^ was the most used in the investigation of sensory processing during this period, followed by the Test of Sensory Function in Infants (TSFI).^26^ The two instruments are considered valid and reliable in the literature. The TSFI is limited to the assessment of children between 4 and 18 months of age, and ITSP evaluates children from birth to 36 months; however, the two instruments differ in the essence of the assessment. ITSP is a questionnaire for parents, and TSFI is an observational instrument. Therefore, it is difficult to compare the results of the two evaluations.^46^

Six studies^35^ ^,^ ^36^ ^,^ ^37^ ^,^ ^38^ ^,^ ^40^ ^,^ ^41^ investigated the association of sensory processing with development outcomes, and it was observed that there is no consensus in the literature on this issue. Eeles et al.^40^ found the association of altered sensory processing with poorer outcomes in motor, cognitive, and language domains in children at 2 years of age who were born preterm. In a similar research, but with children assessed in preschool, Adams et al.^36^ found an association of sensory processing with executive function in preterm children who presented high frequency of altered sensory processing. The findings are consistent with the results of DeGangi et al.,^47^ ^,^ ^48^ which showed consistent evidence of association of SPD, which was identified during the first and second years of life, with deficits in motor, cognitive, and languagedevelopment in preschool age.

According to White et al.,^49^ manifestations of SPD hinder child’s adaptation to environmental demands, limiting their participation in a child’s play and school and/or social activities. Whereas the participation of children in daily activities is crucial to their perception and interaction with the environment,^50^ it is believed that these limitations can negatively affect the development of motor, cognitive, emotional, and language functions in children with SPD.

Finally, the results of this review suggest that sensory processing issues in preterm infants are caused by a combination of biological and neonatal risks of prematurity. Gestational age, male gender, and presence of white matter lesions were the main factors associated with sensory processing outcome in the studies reviewed.

Chorna et al.^38^ showed that preterm infants who were born at 23 weeks of gestation are more likely to present sensory processing alterations than those who were born at 33 weeks. This result suggests that the risk found for SPD in preterm infants increases according to the degree of neurobiological immaturity at birth.

Rahkonen et al.^35^ found that the atypical “sensation seeking” pattern, identified by ITSP, was more frequent inpreterm children, who presented,in the MRI, loss of periventricular white matter, ventricular dilatation, white matter cystic abnormalities,and/or dilation of the subarachnoid space. This result is consistent with the study of Owen et al.,^51^ which examined the impact of structural abnormalities in white matter on the sensory processing, using magnetic resonance diffusion tensor, known as diffusion tensor imaging (DTI). A significant difference was found between children with SPD and children with typical development; those children with SPD presented more frequent structural abnormalitiesin white matter. These observations show that the white matter structure may serve as biological substrate for SPD.

The association of poorer results of sensory processing with male gender in the revised articles may be a disadvantage related to the phenomenon known as “male disadvantage.”^52^ Studies of neonatal mortality reveal that the male gender presents lower overall development speed, particularly of the lung. Pulmonary immaturity increases the incidence of respiratory issues in newborn males, increasing the risk of neonatal morbidity and mortality in this population.^53^ ^,^ ^54^

The results of this review should be interpreted with caution. It was observed that most of these studies described efficiently the populations analyzed, including context delimitation of the search (places and relevant dates), presentation of eligibility criteria, sources and methods of selection of participants, and description of their clinical, demographic, and social characteristics. However, no study reported how the sample size was determined, and this analysis is crucial to ensure the consistency of the results found. Moreover, the evaluation of sensory processing was carried out only by means of questionnaires applied to parents in 62.5% of the studies reviewed.^35^ ^,^ ^36^ ^,^ ^39^ ^,^ ^40^ ^,^ ^41^Although the obtained information provided important description of the child’s sensory processing during activities of daily living, the responses may vary according to parent’s reading skills and interpretation of the questionnaire items, which reduces the degree of evidence of the results.

It is also worth noting that only 50% of studies^36^ ^,^ ^37^ ^,^ ^39^ ^,^ ^42^ reported comparisons of children born prematurely with peers who were born at term. The remaining studies^35^ ^,^ ^38^ ^,^ ^40^ ^,^ ^41^ presented comparisons with normative data established by the assessment tool applied; this may be an issue when participants differ from normative data, for example, in relation to socioeconomic status.

A possible limitation of this review was the exclusion of gray literature - term used to refer to any source of information that is not indexed for publication databases, such as conference abstracts or government reports.^55^ The reason for exclusion was mainly because these type of publication is not peer reviewed, which is an important process that contributes to the study consistency. In addition, only studies published in English, Spanish, and Portuguese were reviewed in this study.

## CONCLUSIONS

Although few studies have currently investigated the sensory processing of children born preterm, this review suggests that prematurity has negative impact on sensory processing in children during childhood. Gestational age, male gender, and white matter lesions appear as risk factors for SPD in preterm infants. The impairment in the ability to receive sensory information and to integrate and adapt to them appears to interfere negatively in motor, cognitive, and language development in this population; however, there is no consensus in the literature on this issue.

The feasibility of the identification of sensory processing issues in the early years of life should be highlighted, a s it favors early referral for intervention. The lack of national studies or of those published in local journals on the theme is clear. Furthermore, the need for longitudinal studies to investigate the prevalence, persistence, and potential consequences of SPD in preterm infants in the medium and long terms should be highlighted.
